# Highly divergent mussel lineages in isolated Indonesian marine lakes

**DOI:** 10.7717/peerj.2496

**Published:** 2016-10-13

**Authors:** Leontine E. Becking, Christiaan A. de Leeuw, Bram Knegt, Diede L. Maas, Nicole J. de Voogd, Abdunnur, Iwan Suyatna, Katja T.C.A. Peijnenburg

**Affiliations:** 1 Department of Environmental Science, Policy, and Management, University of California, Berkeley, CA, USA; 2 Department of Marine Animal Ecology, Wageningen University & Research, Wageningen, The Netherlands; 3 Department of Marine Biodiversity, Naturalis Biodiversity Center, Leiden, The Netherlands; 4 Institute for Biodiversity and Ecosystem Dynamics, University of Amsterdam, Amsterdam, The Netherlands; 5 Faculty of Fisheries and Marine Sciences, Mulawarman University, Samarinda, East Kalimantan, Indonesia

**Keywords:** Phylogeography, East Kalimantan, Anchialine, Geometric morphometrics, Mytillidae, Speciation

## Abstract

Marine lakes, with populations in landlocked seawater and clearly delineated contours, have the potential to provide a unique model to study early stages of evolution in coastal marine taxa. Here we ask whether populations of the mussel *Brachidontes* from marine lakes in Berau, East Kalimantan (Indonesia) are isolated from each other and from the coastal mangrove systems. We analyzed sequence data of one mitochondrial marker (Cytochrome Oxidase I (COI)), and two nuclear markers (18S and 28S). In addition, we examined shell shape using a geometric morphometric approach. The Indonesian populations of *Brachidontes* spp. harbored four deeply diverged lineages (14–75% COI corrected net sequence divergence), two of which correspond to previously recorded lineages from marine lakes in Palau, 1,900 km away. These four lineages also showed significant differences in shell shape and constitute a species complex of at least four undescribed species. Each lake harbored a different lineage despite the fact that the lakes are separated from each other by only 2–6 km, while the two mangrove populations, at 20 km distance from each other, harbored the same lineage and shared haplotypes. Marine lakes thus represent isolated habitats. As each lake contained unique within lineage diversity (0.1–0.2%), we suggest that this may have resulted from *in situ*divergence due to isolation of founder populations after the formation of the lakes (6,000–12,000 years before present). Combined effects of stochastic processes, local adaptation and increased evolutionary rates could produce high levels of differentiation in small populations such as in marine lake environments. Such short-term isolation at small spatial scales may be an important contributing factor to the high marine biodiversity that is found in the Indo-Australian Archipelago.

## Introduction

The world’s largest concentration of marine biodiversity is situated in the Indo-Australian Archipelago (IAA) (Roberts et al., 2002; Hoeksema, 2007). Numerous factors have been proposed that may account for the high biodiversity within the IAA, including the geological history of the area (Renema et al., 2008), its position downstream of the Pacific (Connolly, Bellwood & Hughes, 2003; Kool et al., 2011), the large area of shallow water habitat during the Pleistocene low sea level stands (Voris, 2000; Hoeksema, 2007), great habitat heterogeneity (Hoeksema, 2007), and large reef area (Bellwood et al., 2005). Generally, the pattern that emerges from phylogeographic studies of marine benthic species in the IAA is that of population genetic structuring at small spatial scales (e.g., Timm & Kochzius, 2008; Barber, Erdmann & Palumbi, 2006; Carpenter et al., 2011; Ludt & Rocha, 2015). These results suggest that dispersal barriers over small spatial scales are important in the structuring of diversity that we see today. However, the nature of dispersal barriers (geographic, environmental, biological) for marine taxa has often remained elusive (e.g., Peijnenburg & Goetze, 2013).

Small peripatric populations such as those in marine lakes provide an opportunity to study marine taxa in isolated environments (Dawson & Hamner, 2005). Marine lakes are anchialine systems: small bodies of landlocked seawater isolated by varying degrees from the surrounding marine environment by means of subterranean channels or fissures in the surrounding rock (Holthuis, 1973; Hamner & Hamner, 1998). A large number (10s–100s) of marine lakes are located in the countries Indonesia and Palau (Dawson et al., 2009; Becking et al., 2011; Becking, De Leeuw & Vogler, 2014). The majority of marine lakes are shallower than 50 m. This means that during the Last Glacial Maximum, when sea levels were approximately 110–140 m lower than modern sea levels (Geyh, Kudrass & Streif, 1979; Voris, 2000), the lakes would have been dry or contained fresh water (Dawson, 2006). Based on the sea level rise, the presumed dates of filling of the lakes with seawater are estimated at 6,000–12,000 years before present (Dawson, 2006; Sathiamurthy & Voris, 2006).

Marine lakes are young environments, yet their biodiversity is distinct from the adjacent sea (Dawson & Hamner, 2005; Becking, Cleary & De Voogd, 2013). Several studies of marine lake fauna suggest high endemism or an abundance of species rare elsewhere (Tomascik & Mah, 1994; Dawson, 2005; Dawson & Hamner, 2005; Azzini et al., 2007; Colin, 2009; Becking et al., 2011; Hoeksema, Tuti & Becking, 2014). Phylogeographic studies of populations of the jellyfish *Mastigias papua* (Dawson, 2005; Dawson & Hamner, 2005), the fish *Sphaeramia orbicularis* (Gotoh et al., 2009) and *Atherinomorus endrachtensis* (Gotoh et al., 2011), and the mussels *Brachidontes* spp. (Goto, Tamate & Hanzawa, 2011) from marine lakes of the islands of Palau show genetic isolation, low genetic diversity, and in the cases of *Mastigias papua* and *Brachidontes* spp., rapid morphological evolution. The unique diversity within the lakes could have two origins, which are not mutually exclusive (Briggs, 2000; Bellwood et al., 2005; Bellwood & Meyer, 2009): (1) it is composed of ancient lineages, which are relicts of earlier sea or anchialine populations or (2) it has resulted from recent divergence of rapidly evolving populations isolated from their ancestral population in the sea. When solely originating from ancient lineages, we expect to find frequent haplotype sharing between populations, and evenly distributed haplotype frequencies within populations. In the latter setting we expect to find genetic signatures of recent population expansion and private haplotypes in each of the populations in the lakes. Outside of Palau, the only phylogeographic study of Indo-Pacific marine lake fauna to date was conducted on the sponge species *Suberites diversicolor* in Indonesia (Becking et al., 2013). This study revealed two deeply diverged lineages and suggested that within one lineage there may have been local diversification in the largest and least connected marine lake known in Indonesia (Kakaban lake in East Kalimantan). Here we expand on these results by studying the phylogeography of co-distributed and common marine lakes species, the mussels *Brachidontes spp.*

Species of the genus *Brachidontes* Swainson, 1840 (Mollusca; Bivalvia; Mytilidae) attach themselves to substrate in and below intertidal areas and can form large mytilid beds (Terranova et al., 2007). *Brachidontes* spp. are broadcast spawners with external fertilization and only disperse during their planktonic larval stage for a duration of up to four weeks (Reunov, Au & Wu, 1999; Monteiro-Ribas et al., 2006; Terranova et al., 2006). However, it is unknown whether *Brachidontes* larvae are able to survive in the subterranean channels connecting marine lakes to the surrounding sea. There is an undescribed species of *Brachidontes* that inhabits many marine lakes and, when present, is generally dominant in terms of space occupation and biomass in these lakes (Tomascik & Mah, 1994; Colin, 2009; Becking et al., 2011). In contrast, this species has not been reported in coastal habitats (non-marine lake) (Colin, 2009; Goto, Tamate & Hanzawa, 2011). A previous study of *Brachidontes* from marine lakes in Palau found two genetically distinct and morphologically differentiated lineages (A & B) that most likely represent different species (Goto, Tamate & Hanzawa, 2011). Moreover, the spatial genetic structure of *Brachidontes* from Palau indicated that the majority of the marine lake populations were highly differentiated from each other, each containing private haplotypes (Goto, Tamate & Hanzawa, 2011). A key gap in our understanding of the evolutionary history of *Brachidontes* populations in marine lakes, is the missing sampling from locations in the adjacent sea (e.g., the mangroves) that presumably represent the ancestral populations of those in the lakes.

In order to study the phylogeography and population connectivity of *Brachidontes* spp. we collected sequence data of two nuclear markers, 18S ribosomal RNA (18S) and 28S ribosomal RNA(28S), and of Cytochrome Oxidase I mitochondrial marker (COI) of mussels from three marine lakes and two mangrove locations in Indonesia. In addition, we examined shell outline shape variation using a geometric morphometric approach to determine morphological differentiation. With these data we address the following questions: 1. Which genetic lineages of *Brachidontes* spp. are present in East Kalimantan, Indonesia; 2. Are the marine lakes of East Kalimantan well connected to each other and to the adjacent sea; 3. Based on diversity levels and genetic signatures of mussels in each lake, do we find evidence of a recent population bottleneck followed by expansion?

## Material and Methods

### Sample collection

**Figure 1:**
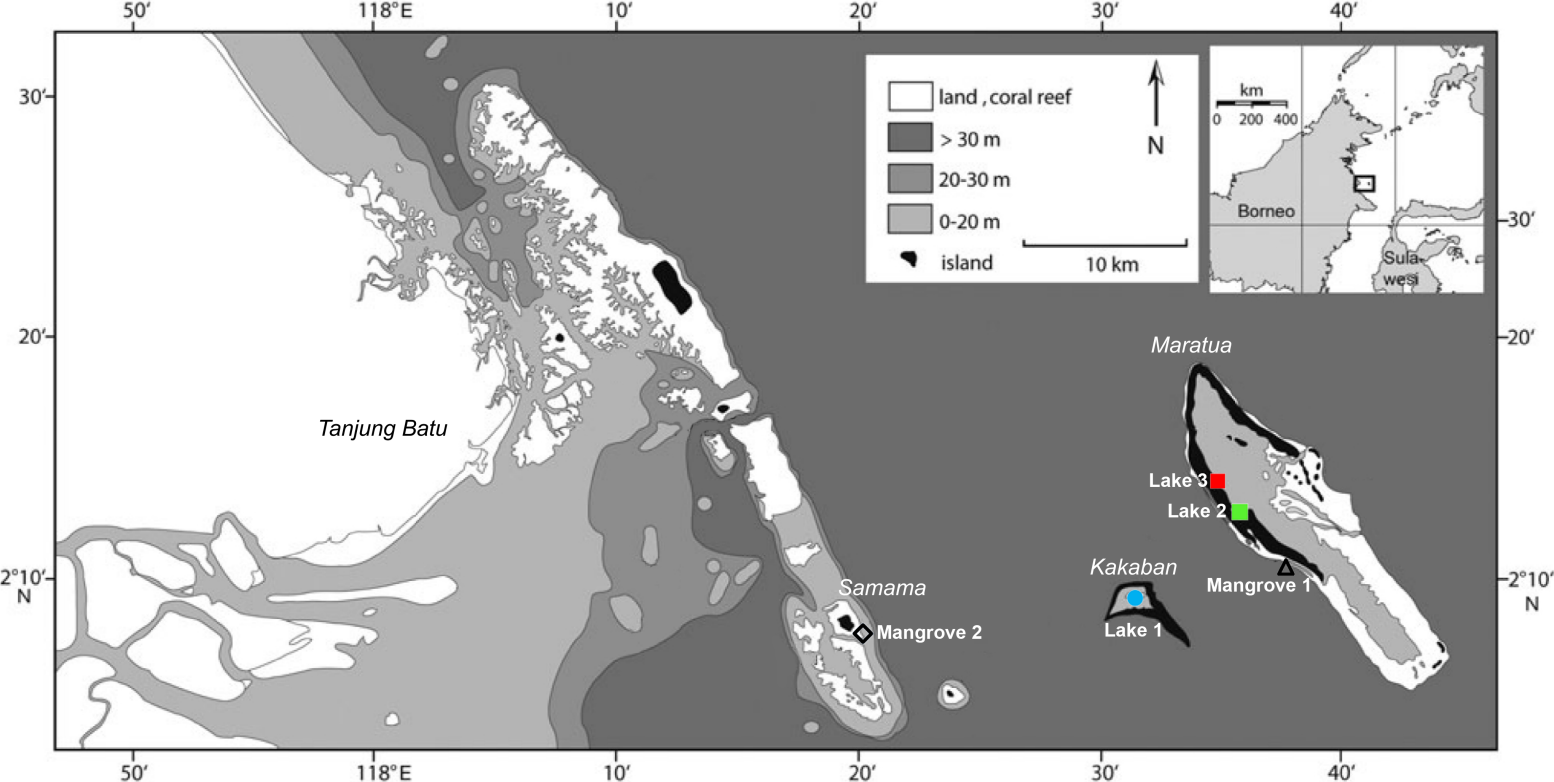
Sample localities of populations of *Brachidontes*spp. in the Berau region, East Kalimantan province. Mussels were samples in three marine lakes (Lake 1, Kakaban Lake; Lake 2, Haji Buang lake; Lake 3, Tanah Bamban Lake) and two coastal mangroves (Mangrove 1, Maratua islands; Mangrove 2, Samama island).

Collection was conducted under research permits #3246/FRP/SM/VII/2012, and #0094/FRP/SM/V/2009 provided by the Indonesian Institute of Sciences (LIPI) and the Indonesian State Ministry of Research and Technology (RISTEK) to LE Becking. The mussels were collected from three marine lakes in Berau, East Kalimantan, Indonesia (Fig. 1 and Table 1): Kakaban lake (Lake 1), Lake Haji Buang (Lake 2), Tanah Bamban lake (Lake 3). Mussels were abundant in the lakes displaying dense mussel-beds along the shorelines, covering rock or mangrove roots. Yet, these species of *Brachidontes* have previously not been found outside of lakes in the Indo-Pacific (Colin, 2009; Goto, Tamate & Hanzawa, 2011). After extensive searching along the coasts of the islands in Berau, we found small populations of mussels in two coastal mangrove systems with a mixture of mangroves species *Rhizophora mucronata* and *Avecinnia alba*, here referred to as Mangrove 1 (Maratua island) and Mangrove 2 (Samama island) (Fig. 1 and Table 1). The mussels from these two areas were burrowed in the sand. All specimens were conserved in 96% laboratory grade EtOH and stored at 4 °C. For a full description of the Berau marine lakes, see Tomascik & Mah (1994) and Becking et al. (2011). The Berau marine lakes have a tidal regime, which is typically delayed (ranging from 20 min to 4 h) and dampened (ranging from 10–80%) compared to the adjacent sea (Becking et al., 2011). Based on the level of tidal dampening and delay, marine lakes can be ranked by their degree of connectivity to the surrounding sea (Hamner & Hamner, 1998), i.e., Lake 1 was the most isolated lake, followed by Lake 2 and Lake 3. Additional COI sequences of *Brachidontes* spp. from Palau were obtained from GenBank (accession numbers: AB465561, AB465566, AB465569). Palau is located at approximately 1,900 km north-east from Berau, East Kalimantan. For a full description of the Palauan marine lakes, see Hamner & Hamner (1998) and Colin (2009).

**Table 1:** Sample localities of *Brachidontes* spp. from Berau, East Kalimantan, Indonesia. Per locality is provided: salinity (parts per thousand, ppt), number of individuals analyzed for morphometric analyses (# morph), number of individuals sequenced for Cytochrome Oxidase I (#COI, number of haplotypes in brackets), haplotype diversity (*h*), percentage nucleotide diversity per population (*π*), mean pairwise differences, all with ± standard deviation (s.d.) obtained with 1,000 bootstrap replicates, and Tajima’s D (*p* < 0.05 indicated by asterisk).

Code	Location	Salinity (ppt)	#morph	#COI	*h* ± s.d.	*π* ± s.d. (%)	Mean pairwise diff ± s.d.	Tajima’s *D*
*Lake*								
Lake 1	Kakaban lake	23–24	87	34 (11)	0.6185 ± 0.0960	0.21 ± 0.16	1.08 ± 0.73	−1.87^∗^
Lake 2	Haji Buang lake	26–28.5	18	25 (10)	0.7267 ± 0.0930	0.33 ± 0.22	1.68 ± 1.02	−2.1^3^
Lake 3	Tanah Bamban lake	26	27	21 (3)	0.3429 ± 0.1236	0.07 ± 0.07	0.36 ± 0.36	−0.80
*Coast*								
MNGR1	Maratua mangrove	33–34	20	7 (5)	0.8571 ± 0.1371	0.72 ± 0.47	3.71 ± 2.13	−1.32
MNGR2	Samama mangrove	33–34	20	16 (9)	0.8833 ± 0.0612	0.47 ± 0.30	2.45 ± 1.4	−1.44

### DNA extraction, gene amplification and sequencing

Posterior adductor muscle tissue was sampled from *Brachidontes* spp. and incubated at room temperature for 24 h in a lysis buffer consisting of 250 mM EDTA, 5% SDS, and 50 mM Tris (pH = 8) (Holland, 1993). Total DNA was purified using DNeasy Blood and Tissue Kit (Qiagen) following the manufacturer’s protocol. COI is an informative marker for high resolution phylogeographic analysis (Lee & Foighil, 2004; Aguirre, Perez & Sirch, 2006; Smietanka et al., 2014). Partial COI was amplified using the CB1F/CO1R primers of Goto, Tamate & Hanzawa (2011) designed for the female type and the jgLCO/jgHCO primers from Geller et al. (2013) for marine invertebrates. Two individuals per lineage were amplified 28S and 18S, using the primers D23F/D6R (Park & Ò Foighil, 2000) and 22F/1789R (Medlin et al., 1988), respectively. These markers were used only on this subset of individuals to assess the phylogenetic context of the lineages within the Mytillidae and we did not expect to find intra-specific or intra-lineage variation with 28S and 18S (Medlin et al., 1988; Park & Ò Foighil, 2000). PCR reactions for all primer pairs were performed in 25 µL volumes containing 0.8 µL 25 mM MgCl_2_, 3 µL dNTP’s (1 mM each), 2.5 µL 10× PCR Buffer (SpheroQ), 0.8 µL BSA (10 mg/mL), 1 µL of both primers (10 µM), 0.2 µL Taq polymerase (5 units/µL) and 1 µL template DNA. The PCR program of the COI primers consisted of an initial denaturation step of 94 °C for 3 m followed by 35 PCR cycles of 94 °C for 45s, 49 °C for 30s and 72 °C for 120s, with a final extension step of 72 °C for 5 m. The PCR program of the 18S and 28S primers was identical to the previously described except for the annealing step at 47 °C for 35s. Quality of PCR products was assessed using gel electrophoresis on 1% agarose gels. If PCR product quality was insufficient, a new PCR reaction was performed in a 25 µL volume containing PCR Beads (Illustra, GE Healthcare) using 21.6 µL ddH_2_O, 0.2 µL of both primers (10 µM) and 3 µL template DNA, and an identical PCR cycling program. PCR products were purified and sequenced by Macrogen Inc. (Korea and The Netherlands).

### Doubly uniparental inheritance (DUI)

Some species of the family Mytilidae display a type of mitochondrial inheritance called ‘doubly uniparental inheritance’ (DUI) (Zouros et al., 1994) in which two different mitochondrial lineages, a male (M-type) and female (F-type) lineage, are present in male individuals (Terranova et al., 2007; Goto, Tamate & Hanzawa, 2011). Male and female mitochondrial lineages can be highly diverged (Saavedra, Reyero & Zouros, 1997; Kenchington et al., 2002). We avoided obtaining M-type sequences by sampling muscle tissue, avoiding gonads, and using specific primers for F-type sequences. With an additional check, the distinction between F- and M-type sequences in our data was made by constructing a phylogeny. We obtained two M-type sequences (Genbank accession numbers KX688100 and KX688101). Subsequently, the analyses in the present paper were done only on the female lineages, which is standard in phylogeographic studies of mussels (e.g., Goto, Tamate & Hanzawa, 2011; Smietanka et al., 2014).

### Genetic data analysis

The bivalve origin of the obtained sequences was verified through BLAST searches (http://blast.ncbi.nlm.nih.gov/Blast.cgi). From the blast results for the 28S and 18S sequences, the most similar sequences were selected and included in the subsequent phylogenetic analysis. Sequences were aligned and handled in CodonCode aligner v4.1.1 (CodonCode Corporation, USA) and DAMBE 5.2.15 (Xia & Xie, 2001; Xia, 2013). The best-fit DNA substitution model was selected by the Akaike Information Criterion deployed in jMODELTEST v. 2.1.6 (Darriba et al., 2012). These models (COI: Hasegawa-Kishino-Yano (Hasegawa, Kishino & Yano, 1985); 18S: Kimura 2-parameter (Kimura, 1980); 28S: Tamura-Nei (Tamura & Nei, 1993); all with a discrete gamma distribution) were used for subsequent maximum likelihood phylogeny inferences implemented in MEGA v6.06 (Tamura et al., 2011) using a heuristic search with 1,000 bootstrap replicates. The resulting phylogenetic trees were visualized in FigTree v1.4.0 (Rambaut, 2009). Separate maximum likelihood analyses were carried out for each marker. Net nucleotide distances between lineages were calculated in MEGA v6.06 using the models obtained with jMODELTEST. Net nucleotide divergence corrects for discrepancies between gene divergence and population divergence due to ancestral polymorphism in populations (Edwards & Beerli, 2000), since it subtracts the average within-group divergence from the observed between-group estimate. Estimates of genetic variation in samples pooled per location were obtained as haplotype diversities *h* (Nei, 1987), nucleotide diversities *π* (Tajima, 1983; Nei, 1987), and mean pairwise differences using Arlequin version v.3.5 (Excoffier & Lischer, 2010). In order to create a haplotype network, separate maximum likelihood tree analyses were carried out for each lineage (following the description above), and the resulting phylogenetic trees were used as input for Haploviewer (Salzburger, Ewing & Von Haeseler, 2011).

Two methods were used to test for signatures of recent population expansion. First, Tajima’s *D* tests of selective neutrality (Tajima, 1983) were carried out in Arlequin to compare the observed numbers of pairwise nucleotide differences between haplotypes in a sample with expectations under an infinite-sites model of sequence evolution, and under assumptions of selective neutrality and stable population size. Significance was tested by generating 10,000 random permutations. Second, mismatch distributions were calculated in Arlequin (Excoffier & Lischer, 2010) and DnaSP (Rozas et al., 2003) to test for signatures of demographic expansion and to test the null hypothesis of population growth. The observed distribution of pairwise differences between sequences was compared with a theoretical distribution, as expected under a sudden expansion model (Rogers & Harpending, 1992) computed in DnaSP (Rozas et al., 2003). To test the fit of the sudden-expansion-model, the sum of squared deviations (SSD) between the observed data and theoretical model was calculated in Arlequin. Harpending’s raggedness index (rg) was used to determine the smoothness of the observed mismatch distribution, which can be used to distinguish between expanded and stationary populations (Harpending et al., 1993). Expanding populations generate smooth and unimodal distributions, while more stationary populations the mismatch distribution becomes more ragged and erratic. The value of the raggedness index will be low and non-significant in expanding populations, while it is usually high and significant in stationary populations (Harpending, 1994; Harpending et al., 1993).

### Morphometric analysis

*Brachidontes spp.* mussels were photographed in a standardized orientation for geometric morphometric analyses. In total 172 digital images were stored as Nikon RAW format (.nef) and converted to 3,008 × 2,000 pixel JPEG images using Photoshop 5.0 (Adobe). JPEG images were sampled into TPS files using the program *tpsUTI*L (Rohlf, 2010a). Shell outlines were used to capture variation in shell shape of *Brachidontes* spp. We used a sliding semi-landmark analysis, in which semi-landmarks are allowed to slide along the outline of a shell in order to find the position that optimally matches the positions of corresponding semi-landmarks in a consensus specimen (Bookstein, 1997; Adams, Rohlf & Slice, 2004). Shell outlines were drawn as curves and digitized as 68 semi-landmarks at equal distance using *tpsDig* (Rohlf, 2010b), using the beak of the mussel (umbo, see Fig. S1) as a standardized starting point for drawing an outline. A “sliders file” indicating sliding semi-landmarks was made using *tpsUtil* (Rohlf, 2010a). To standardize for size and orientation we used *tpsRelw* (Rohlf, 2010c) with Generalized Procrustes Superimposition (Rohlf, 1999). Residuals from the superimposition were analysed with the thin-plate spline interpolating function, producing principal warps, followed by relative warp (RW) analysis. The program *TpsRelw* was used to obtain centroid size (Bookstein, 1991) and RW scores for each individual. RW axes are analogous to the eigenvectors of principal component analysis, which combine the major patterns of shell shape variation in the data. Repeatability of RW axes was tested using regression analysis and a non-parametric analysis of similarity in PAST 2.11 (Hammer, Harper & Ryan, 2001) of RW scores extracted from 17 specimens of *Brachidontes* spp., which were independently photographed. RW axes were considered repeatable when they showed a non-significant and close to zero *R*-value in the analysis of similarity and a strong (*r* > 0.7) and significant correlation. Only repeatable relative warp axes were included in further analyses of shell shape variation. Correlations of RW scores with centroid size were tested, and if significantly correlated, we used residuals of the regression in analyses of shell shape. Significant differentiation between populations was tested using a non-parametric analysis of similarity (ANOSIM, 10,000 randomizations) (Clarke, 1993) based on Euclidian distance as implemented in PAST v2.11 (Hammer, Harper & Ryan, 2001).

## Results

### Genetic analysis

**Figure 2:**
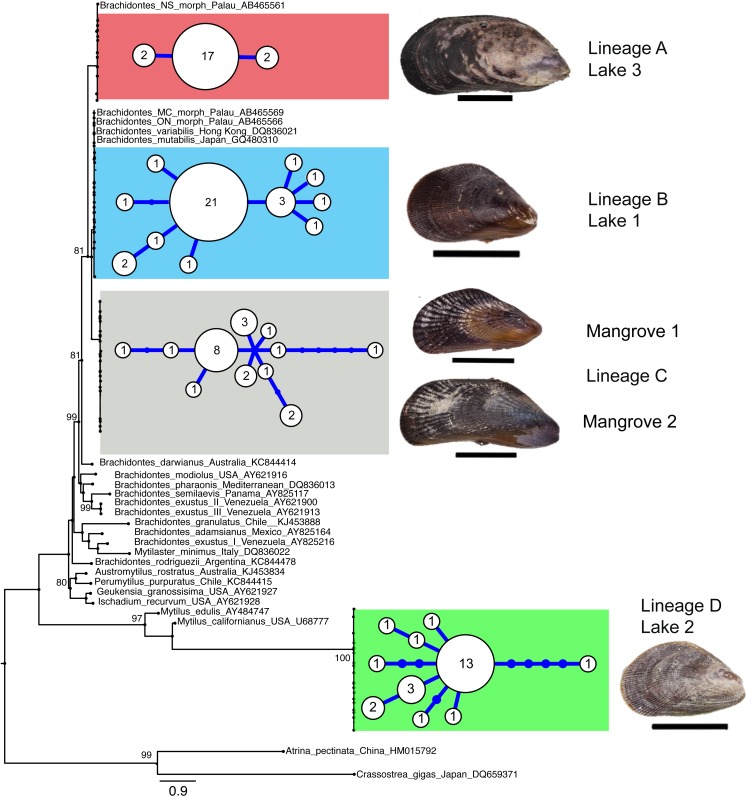
Genetic lineages of mussels in marine lakes. Mid-point rooted Maximum likelihood phylogram of partial Cytochrome Oxidase I sequences of *Brachidontes* spp. sampled from Indonesia (this study) and Palau (Gotoh et al., 2011), including haplotype networks per location and image of representative specimen per lineage (black scale bars indicate 1 cm). See Fig. 1 and Table 1 for details of location. The sequences from the present study from Indonesia are indicated by location codes (see Table 1; color codes correspond to Fig. 1). Sequences of *Brachidontes* from Palau marine lakes are from the study Gotoh et al. (2011) and are represented with the morphological type, and Genbank accession number. Only maximum likelihood support of >70% are indicated. Scale bar indicates substitutions/site. To the right of the phylogram, minimum spanning networks are shown of haplotypes found in the Indonesian populations of the present study. Each circle represents a single haplotype and its diameter is approximately proportional to the number of individuals carrying that haplotype (number provided in circle), with the smallest circle representing a single individual. Lines connecting haplotypes represent one base substitution between two haplotypes with additional mutational steps indicated by dots. The corrected genetic distance between the lineages in provided in Table 2.

A total of 103 COI sequences of 516 bp (aligned length) were collected, resulting in 36 F-type haplotypes (Genbank accession numbers KX346179 –KX346214). No stop codons, indels or double peaks were observed in these sequences. The COI sequences represented four major lineages that were strongly supported by maximum likelihood analyses and these lineages corresponded with locality (Fig. 2 and Table 2). Two of these four divergent lineages correspond to ‘lineage A’ and ‘lineage B’ from the marine lakes in Palau (Goto, Tamate & Hanzawa, 2011) which naming we adopt (Fig. 2). In addition, two new lineages were detected, which we have named C & D. In the present study lineage A was present in Lake 3 (Tanah Bamban Lake), while lineage B was present in lake 1 (Kakaban Lake), lineage C was present in both mangrove localities (Samama and Maratua), and lineage D was present in lake 2 (Haji Buang Lake). A subset of individuals of Lineage A–D were sequenced for a 976 bp long fragment of 28S (aligned length, Genbank accession number KX346215 –KX346217) and 18S (1,323 bp, KX346218 and KX346219) (Figs. S2 and S3) this resulted in identical sequences for Lineage A–C, which fall in the clade *Brachidontes* s.s. established by Trovant et al. (2015) and are most closely related to *Brachidontes* spp. from marine lakes in Palau. Though the lineages A & B occur in both Indonesia and Palau, not one haplotype was shared between the different marine lakes populations of *Brachidontes* spp. from Indonesia and Palau. The mussels from Lake 3 are 1.8% diverged from Lineage A from Palau, and the mussels from Lake 1 are 0.9–2.8% diverged from Lineage B from Palau (Table 2). Lineage D was the most divergent from the other three lineages: 67–74% corrected divergence in COI (Table 2), 15% in 28S, and 5% with 18S. Blast results of COI, 28S, and 18S of Lineage D did not show high similarity (all less than 90%) with any species in the database.

**Table 2:** Net nucleotide divergence based on partial Cytochrome Oxidase I sequences of *Brachidontes* spp. mussels from marine lakes and mangroves in Indonesia and Palau. Main lineages (A, B, C, D) provided in brackets. Standard deviations are shown above the diagonal. Three morphotypes from Palauan marine lakes were compared: MC- and ONmorphotypes of lineage B, and NS-morphotype from lineage A (see Gotoh et al., 2011 for further details of morphotypes).

	Lake 1	Lake 2	Lake 3	MNGR 1	MNGR 2	Palau_MC	Palau_NS	Palau_ON
Lake 1 (B)		0.088	0.025	0.019	0.022	0.005	0.026	0.005
Lake 2 (D)	0.737		0.091	0.077	0.081	0.085	0.090	0.084
Lake 3 (A)	0.194	0.741		0.030	0.033	0.025	0.006	0.026
MNGR1 (C)	0.137	0.668	0.223		0.000	0.019	0.030	0.020
MNGR2 (C)	0.157	0.701	0.254	0.000		0.022	0.033	0.022
Palau_MC morph (B)	0.009	0.709	0.192	0.132	0.152		0.026	0.003
Palau_NS morph (A)	0.195	0.744	0.018	0.229	0.260	0.189		0.027
Palau_ON morph (B)	0.028	0.648	0.141	0.138	0.158	0.020	0.144	

Each lake harbored only one lineage (Fig. 2), while no lineages were shared among lakes in East Kalimantan. In contrast, the two mangrove locations (Mangrove 1 & 2) shared the same lineage (C) and shared haplotypes. The nucleotide diversity, haplotype diversity and mean pairwise difference (Table 1) were lowest in the lakes (*π*: .1–0.2%; *h*: 0.3–0.7%; pairwise difference: 0.4–1.7) and highest in the open populations of the mangroves (*π*: 0.5–75%; *h*: 0.9; pairwise difference: 2.5–3.7).

### Demographic analysis

**Figure 3:**
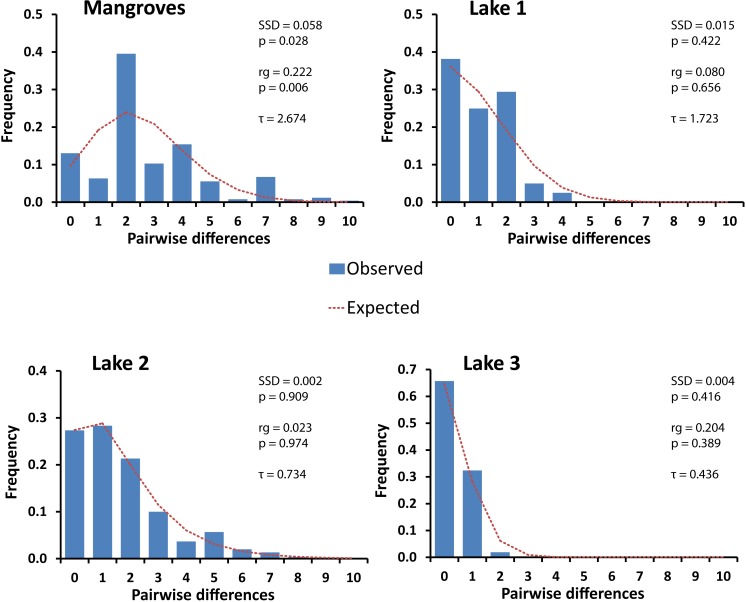
Mismatch distributions of pairwise Cytochrome Oxidase I sequence differences for populations of *Brachidontes* spp. in marine lakes and mangrove systems in Berau, Indonesia. Expected distributions under a model of constant population size and under a sudden population expansion model are indicated as computed in DnaSP (Rozas et al., 2003). Sum of squared deviations (SSD) between the observed data and the theoretical model of demographic expansion are shown on the right, as well as the raggedness index (rg), and tau (*τ*).

In each lake the haplotype network was starlike shape with a common central haplotype different from most of the other haplotypes by one or a few nucleotide substitutions (Fig. 2). Mismatch distributions were clearly unimodal for the three lake populations (Fig. 3), typical of populations that have undergone a recent bottleneck in population size (e.g., during colonization events) followed by rapid population expansion (Slatkin & Hudson, 1991; Rogers & Harpending, 1992).

This pattern was congruent with the raggedness index, which was low and non-significant, and with the Tajima’s *D* statistic, which for lakes 1, 2, and 3 was negative and for lakes 1 and 2 differed significantly from expectations under a neutral model of evolution assuming constant population size (Table 1). By contrast, the mangroves did not have a single dominant haplotype and displayed a multimodal mismatch distribution (Fig. 3), which significantly deviated from an expansion model (Table 1). The mean number of pairwise differences among the mangroves is higher than that in the lakes and assuming a constant mutation rate, this suggest that if a population bottleneck has occurred in the mangroves, this would have happened longer ago in the mangroves than in the lakes (Fig. 3 and Table 1). A significant Tajima’s *D* could indicate a recent population expansion, but could also be a result of a selective sweep (Tajima, 1989a; Tajima, 1989b; Aris-Brosou & Excoffier, 1996; Peck & Congdon, 2004). Likewise, a unimodal mismatch distribution can also be the result of a selective sweep. Here we take a comparative approach utilizing the same markers in species with a very similar biology. Hence, we assume that differences in genetic signatures between samples are most likely due to differences in demographic histories, and not selection on the markers.

### Morphometric analysis

**Figure 4:**
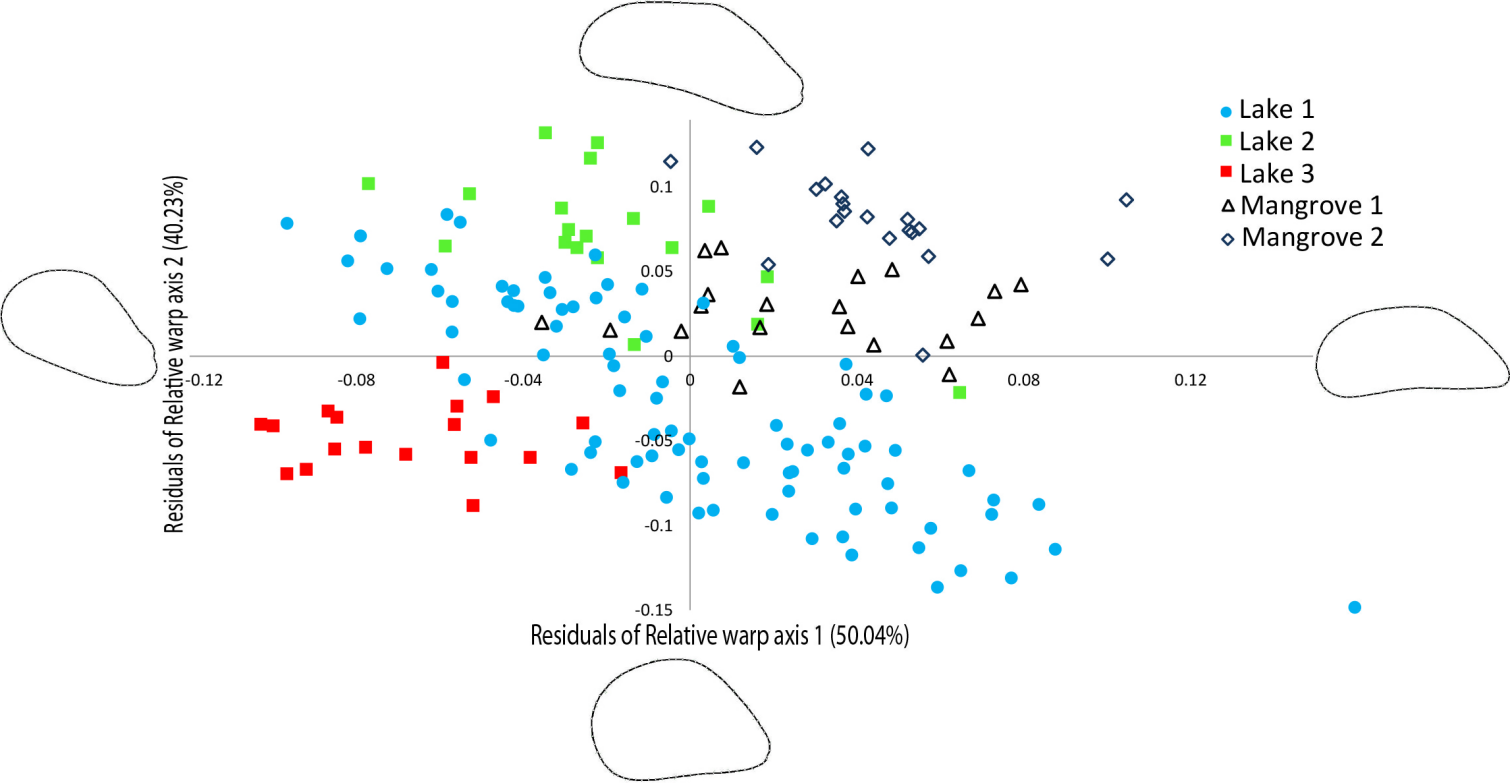
Geometric morphometric analysis of *Brachidontes* spp. shell shape. Ordination of shell outline shape variation in *Brachidontes* spp. mussels from marine lakes and mangroves in Berau, Indonesia (sample locations are detailed in Table 1, shape and color of symbols correspond to Fig. 1). Ordination of data in the plane identified by relative warp (RW) axis 1 and 2, together explain 90.27% of the variance. Corresponding thin-plate splines of the most positive and negative deformations along the axes are shown. Data for both axes are residuals of a regression of RW-scores against centroid size.

The relative warp (RW) axes 1–2 were significantly repeatable, explaining 90.27% of the total observed variation in shell outline shape (Fig. 4). The residuals of the RW axes 1 and 2 were used to correct for the correlation with centroid size (RW1: *R*^2^ = 0.4095, *p* = 0.0001, RW2: *R*^2^ = 0.0285, *p* = 0.0296). Figure 4 shows a scatter plot of the individuals along the two first axes of variation in shell outline shape, indicating how 90.27% of total observed variation in shell outline is distributed along these axes. Variation in shell outline was found in the position of the umbo relative to the longitudinal axis of the shell (axis 1) and the shell length to width ratio (axis 2). Overall shell shape variation among populations was highly significant (One-Way Permanova, global F =21.68, *p* = 0.0001) and all pairwise comparisons between locations showed significant differences (Table 3). A brief morphological description of each of the lineages is provided in S5.

**Table 3:** Analysis of similarity of shell outline shape for *Brachidontes* spp. based on relative warp axes 1–2. *R*-values are shown, all populations are significantly different from each other (*p* < 0.01 using sequential Bonferroni corrected *p*-values).

	Lake 1	Lake 2	Lake 3	MNGR1
Lake 2	19.22			
Lake 3	10.4	80.21		
MNGR1	95.48	21.79	99.44	
MNGR 2	28.17	19.92	21.92	25.64

## Discussion

### Divergent *Brachidontes* lineages in Indonesia and Palau

We detected four mitochondrial female-type lineages based on COI sequences in *Brachidontes* spp. mussels sampled in marine lakes and adjacent mangrove habitats in Indonesia, viz. lineage A, B, C and D (Fig. 2). Two major lineages (A and B) are closely related to the lineages A and B, respectively, reported by Goto, Tamate & Hanzawa (2011) for *Brachidontes* spp. from marine lakes in Palau. Considering the degree of genetic divergence between the four lineages (14–74%), and corresponding morphological differentiation, the lineages probably represent four distinct species. Furthermore, the genetic distances between lineages A–D are comparable to those reported between (cryptic) species of other mussels (Lee & Foighil, 2004; Lee & Foighil, 2005; Terranova et al., 2007; Cunha et al., 2011) Lineage A–C fall within the clade *Brachidontes* s.s. established by Trovant et al. (2015) and are closely related to *Brachidontes* spp. from marine lakes in Palau, as well as *Brachidontes variabilis,* and *Brachidontes mutabilis* (Fig. 2, Fig. S3). In contrast, Lineage D does not fit within the three proposed clades of Brachidontinae and probably represents a distinct genus.

The patterns of COI genetic differentiation of the *Brachidontes* spp. lineages were largely congruent with shell outline shape differentiation. There was, however, one exception: the two mangroves are genetically part of the same lineage and share haplotypes, yet morphometrically they differ significantly. Morphology can provide a proxy for underlying genetic variation, adaptive evolution, as well as phenotypic plasticity related to environmental regimes (e.g., Luttikhuizen, Drent & Baker, 2003; Dawson & Hamner, 2005; Mariani, Peijnenburg & Weetman, 2012; Burridge et al., 2015). It is likely that part of the morphometric variation in shell outline shapes of *Brachidontes* spp. mussels are the result of highly distinct environmental conditions at each locality (Table 1). Worldwide 31 species of *Brachidontes* have been described, but the phylogenetic positions of the different species within this genus remains unclear with several reports of the occurrence of cryptic species (Lee & Foighil, 2004; Lee & Foighil, 2005; Aguirre, Perez & Sirch, 2006). Further taxonomic review of these Indo-Pacific lineages is clearly needed.

### Isolated populations in Indonesian marine lakes

In our study we found that each marine lake harbors a population of mytilid bivalves, but each lake is dominated by a different species that is morphologically and genetically distinct. It is remarkable that the lakes are at such short distance from each other (2–6 km), yet do not share lineages, while the two mangrove locations 20 km apart do. The overall high level of variation is most pronounced in the mangroves, which is interpreted as evidence of a larger population, more stable demographic history and/or more connected systems than the lake systems. Dawson & Hamner (2005) found that the genetic distance between marine lake and lagoon populations of the jellyfish *Mastigias papua* in Palau was highly correlated with the degree of physical isolation of the lake to the adjacent sea, and not the actual geographic proximity of populations to each other. Isolation of marine lake populations may be the result of strong physical isolating barriers as well as different selective environmental regimes in the lakes. The subterranean channels that connect each lake with the surrounding sea may provide a formidable dispersal barrier for propagules. Alternatively, a propagule may be able to enter but may not be able to survive due to the environmental regime within the lake or competition with resident founder lineages/species. The pattern that is almost consistently seen in a variety of taxa (jellyfish, fish, and bivalves) is that each lake harbors a single lineage per taxon (Dawson, 2005; Dawson & Hamner, 2005; Gotoh et al., 2009; Goto, Tamate & Hanzawa, 2011). An explanation for this pattern may be fierce inter-lineage competition, as would be expected for closely related species that occupy similar niches. This is further supported by the observation that lakes either contain mussels or oysters as the dominant bivalves, rarely both (Becking et al., 2011). It could be that priority effects (Alford & Wilbur, 1985; Wilbur & Alford, 1985) provide competitive advantage to whichever lineage colonizes a marine lake first The dominance of a lineage would be largely stochastic, likely dictated by the chance arrival of propagules (Gillespie & Baldwin, 2009): whichever mussel lineage arrives first is the one that fills that niche space. Paleoecological records (e.g., sediment cores with bivalve shells) could potentially verify which morphotypes have been dominant historically.

Each lake appears to show the genetic signature of a recent bottleneck in population size followed by an expansion such as would be expected in founder populations (Slatkin & Hudson, 1991; Rogers & Harpending, 1992). We assume that the floodwaters that filled the lakes during the Holocene sea level rise allowed for independent, chance colonization of lakes by propagules from the surrounding sea and that these propagules were the progenitors of the present-day populations (Dawson & Hamner, 2005; Dawson, 2006). The deep divergences that are observed between the lineages in the mussels are probably ancient lineages that have taken refuge in the lakes. We suggest, however, that the within lineage diversity that is unique to each lake may have resulted from *in situ* divergence during the 6,000–12,000 years that the lakes have existed. This scenario would imply relatively rapid evolutionary rates, but such rates are not uncommon for recently diverged taxa (e.g., Genner et al., 2007; Ho et al., 2011) and have been recorded generally in mtDNA of mussels (e.g., Lee & Foighil, 2004; Trovant et al., 2015). Combined effects of stochastic processes (e.g., founder effects), local adaptation and increased evolutionary rates could produce high levels of differentiation in small isolated populations such as in marine lake environments (Dawson & Hamner, 2005; Ho et al., 2011). The patterns of genetic variation found so far in marine lake populations of *Mastigias papua* (Dawson & Hamner, 2005) * Brachidontes* spp (Goto, Tamate & Hanzawa, 2011; this study), *Suberites diversicolor* (Becking et al., 2013), *Sphaeramia orbicularis* (Gotoh et al., 2009) are generally consistent with taxa evolving in isolation in peripatric environments, such as islands or satellite lakes of ancient rift lakes in Africa (e.g., Genner et al., 2007; Emerson & Gillespie, 2008; Chen & He, 2009; Rosindell & Phillimore, 2011). The role of marine lakes in supporting endemism in the IAA region may be through enhanced survival of endemics (relicts from earlier anchialine systems), with the possibility of local population divergence that may lead to speciation.

The islands of Samama, Kakaban, Maratua are part of the Berau Marine Protected Area that was formed in 2005 and Kakaban island with the large marine lake (Lake 1) is planned to be proposed for a UNESCO World Heritage Site (Gunawan & Visser, 2012; Vermaat, Estradivari & Becking, 2012). The implication of our results for marine conservation is that each lake is barely connected by effective dispersal and must be viewed as unique and isolated entities.

##  Supplemental Information

10.7717/peerj.2496/supp-1 Figure S1 Overview of semi-landmarks used for morphometric analyses Shell outlines were drawn as curves and digitized as 68 semi-landmarks at equal distance using *tpsDig* (Rohlf, 2010b), using the beak of the mussel (umbo, larger dot) as a standardized starting point for drawing an outline. Black scale bars indicates 1 cm.

10.7717/peerj.2496/supp-2 Figure S2 28S phylogram of marine lake *Brachidontes*spp. and other Mytillidae Mid-point rooted Maximum likelihood phylogram of 28S sequences of *Brachidontes* spp. sampled from Indonesian marine lakes and mangroves, compared with species of the family Mytillidae (Genbank accession numbers provided behind species names). Blue-highlighted samples are sequences from the current study. Samples from lake 1 & 3, mangrove 1 & 2 had identical sequences. Lake 2 represents a distant clade.

10.7717/peerj.2496/supp-3 Figure S3 18S phylogram of marine lake *Brachidontes* spp and other Mytillidae Mid-point rooted Maximum Likelihood phylogram of 14 sequences of 18S sequences of *Brachidontes* spp. sampled from Indonesian marine lakes and mangroves, compared with other species of Mytillidae (Genbank accession numbers provided behind species names). Blue-highlighted samples are sequences from the current study. Samples from lake 1 & 3, mangrove 1 & 2 had identical sequences with a sample for Brachidontes from a marine lake in Palau. Lake 2 represesnts a distant clade.

## Additional Information and Declarations

### Field Study Permissions

The following information was supplied relating to field study approvals (i.e., approving body and any reference numbers): The Indonesian Institute of Sciences (LIPI) and the Indonesian State Ministry of Research and Technology (RISTEK) provided research permits to LEB (permit numbers: 3246/FRP/SM/VII/2012, and 0094/FRP/SM/V/2009).

### DNA Deposition

The following information was supplied regarding the deposition of DNA sequences: GenBank: KX688100, KX688101, KX346179 –KX346219.
