# Supplementary figures and images for: Highly divergent mussel lineages in isolated Indonesian marine lakes
Source: PeerJ. 2016 Oct 13;4:e2496. doi: 10.7717/peerj.2496 (PMC5068364; doi:10.7717/peerj.2496)

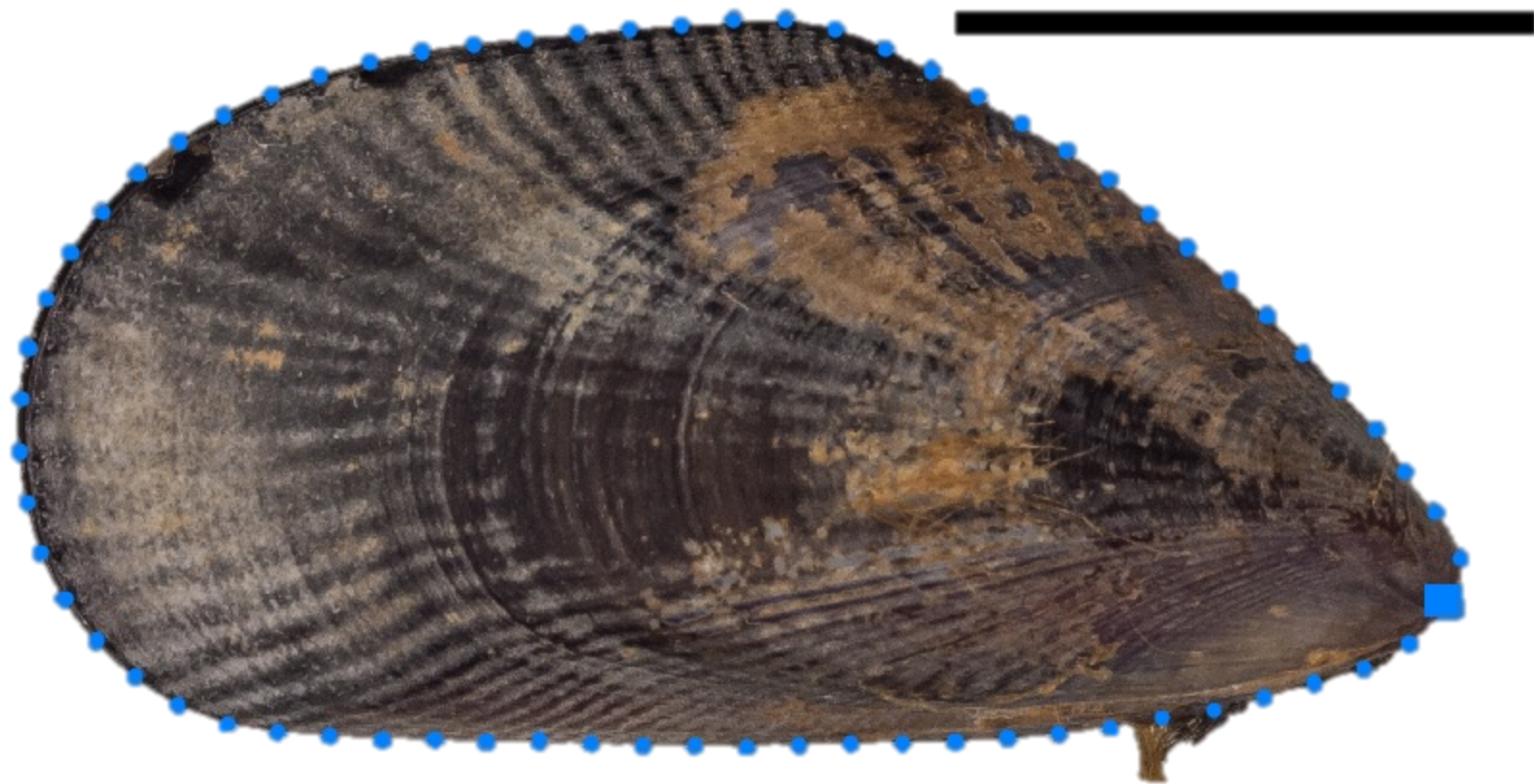

Supplement: Figure S1 — Shell outlines were drawn as curves and digitized as 68 semi-landmarks at equal distance using tpsDig (Rohlf, 2010b), using the beak of the mussel (umbo, larger dot) as a standardized starting point for drawing an outline. Black scale bars indicates 1 cm. [file peerj-04-2496-s001.pdf]

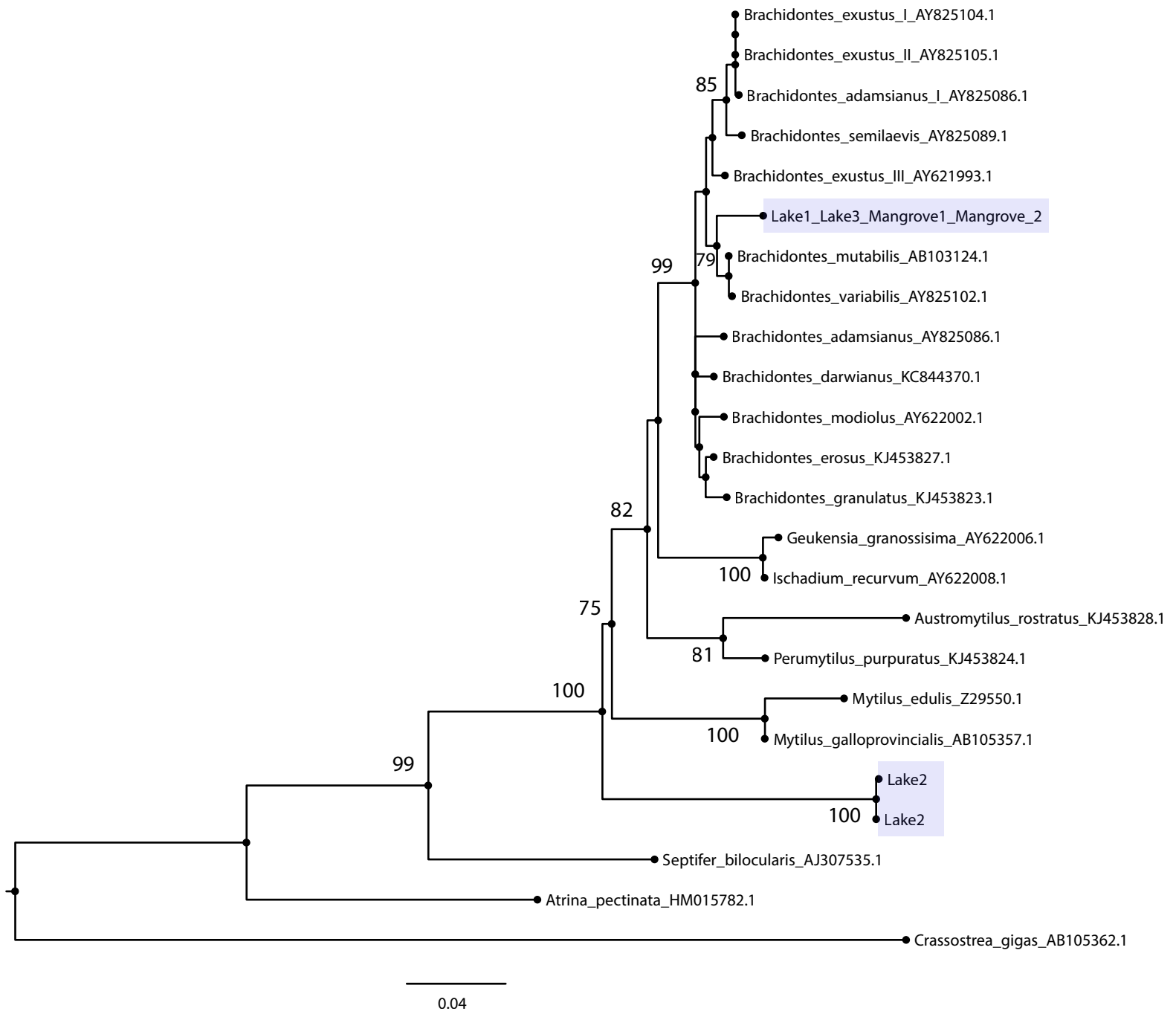

Supplement: Figure S2 — Mid-point rooted Maximum likelihood phylogram of 28S sequences of Brachidontes spp. sampled from Indonesian marine lakes and mangroves, compared with species of the family Mytillidae (Genbank accession numbers provided behind species names). Blue-highlighted samples are sequences from the current study. Samples from lake 1 & 3, mangrove 1 & 2 had identical sequences. Lake 2 represents a distant clade. [file peerj-04-2496-s002.pdf]

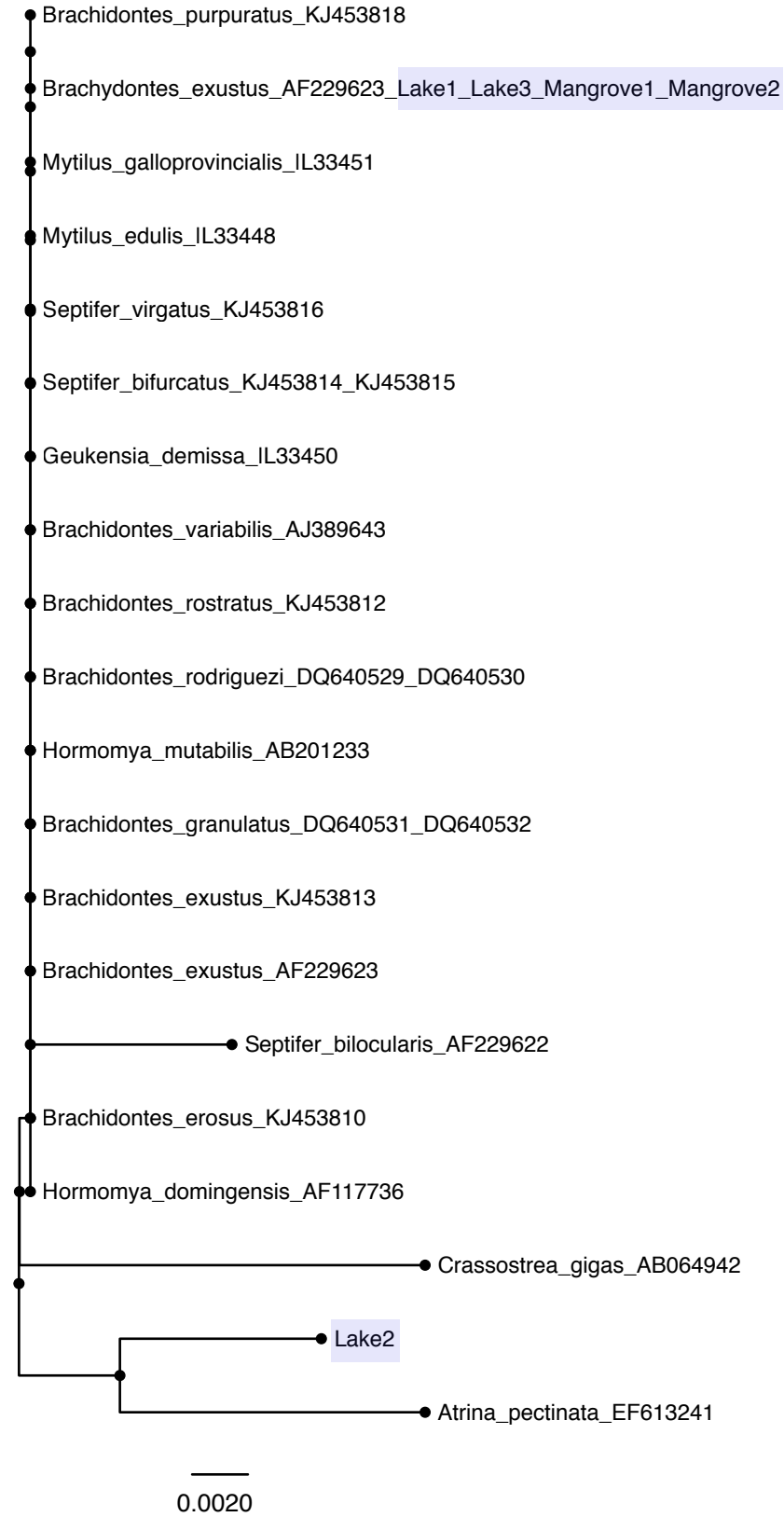

Supplement: Figure S3 — Mid-point rooted Maximum Likelihood phylogram of 14 sequences of 18S sequences of Brachidontes spp. sampled from Indonesian marine lakes and mangroves, compared with other species of Mytillidae (Genbank accession numbers provided behind species names). Blue-highlighted samples are sequences from the current study. Samples from lake 1 & 3, mangrove 1 & 2 had identical sequences with a sample for Brachidontes from a marine lake in Palau. Lake 2 represesnts a distant clade. [file peerj-04-2496-s003.pdf]
